# Career Preference and Factors Influencing Career Choice among Undergraduate Pharmacy Students at University of Khartoum, Sudan

**DOI:** 10.3390/pharmacy10010026

**Published:** 2022-02-07

**Authors:** Ahmed H. Arbab, Yasir A. M. Eltahir, Fatima S. Elsadig, Bashir A. Yousef

**Affiliations:** 1 Department of Pharmacognosy, Faculty of Pharmacy, University of Khartoum, Khartoum 11111, Sudan; ahmed.arbab@uofk.edu; 2 Department of Respiratory Care, College of Applied Sciences, Almaarefa University, Riyadh 11597, Saudi Arabia; Ytaher@mcst.edu.sa; 3 Department of Anatomy, Faculty of Medicine, Kordofan University, Elobeid 51111, Sudan; 4 Faculty of Pharmacy, University of Khartoum, Khartoum 11111, Sudan; Fatimashihab105@gmail.com; 5 Department of Pharmacology, Faculty of Pharmacy, University of Khartoum, Khartoum 11111, Sudan

**Keywords:** career preference, career choice, pharmacy students, Sudan

## Abstract

The pharmacy profession has expanded and adapted to changes in community needs. Although career planning and understanding the determinants of career choice are essential, there remains a lack of studies exploring factors influencing future career plans. This study was conducted to identify career preferences and factors influencing future career choices among undergraduate pharmacy students. A cross-sectional study was carried out at the Faculty of Pharmacy, University of Khartoum. A self-administered questionnaire was used to collect data from randomly selected participants. Out of 220 respondents, 85.9% were females. The average age of the respondents was 21.7 ± 1.5 years. Clinical pharmacy was selected as the most preferred future career domain (30%), followed by academia and research (12%), the pharmaceutical industry (11%), and community pharmacy (10.5). Approximately 20% of participants reported a preference for moving abroad for work. Regarding factors influencing future career domain choice, participants ranked training in the workplace (80%) and curriculum content (70%) as the top faculty-related factors, while interaction with practicing pharmacists (71.8%) and salary (78%) were the major personal-related and job-related factors. This study emphasized the importance of understanding job preferences and the factors influencing career choice, and could be useful in ensuring a future balance between professional domains and meeting society’s evolving expectations.

## 1. Introduction

The pharmacy profession has transformed and adapted itself to changes in the health care system and social needs. It has expanded from a drug-focused profession to include patient care and service-driven professions [1,2]. Until the twentieth century, pharmacists’ responsibilities were limited to compounding, quality control, and dispensing. In 1997, the World Health Organization (WHO) introduced the ‘seven-star pharmacist’ concept, covering the roles each pharmacist must perform: caregiver, decision-maker, communicator, manager, lifelong learner, teacher, and leader [3]. Two criteria, ‘researcher’ and ‘entrepreneur’, were added later, which culminated in the ‘nine-star pharmacist’ concept [4]. In 2000, the seven-star pharmacist concept was adopted in the International Pharmaceutical Federation (FIP) policy statement on good pharmacy education practice [5]. Currently, in addition to their classical roles as drug specialists, pharmacists work in multi-disciplinary settings to deliver pharmaceutical care. As a part of the health care system, their roles involve patient-oriented services, patient education, and counseling about medication and patients’ quality of life [6].

In Sudan, the pharmacy profession is locally governed by the Sudan Medical Council and Directorate General of Pharmacy. Undergraduate pharmacy education lasts for 5 years, and the student acquires a Bachelor of Pharmacy (B. Pharm) degree upon completion. This is followed by a mandatory one-year internship in a governmental pharmacy sector, and then a license is issued after passing an exam conducted by the Sudan medical council. About 67% of the pharmacist workforce is employed in private retail pharmacies; 19% are employed in the public sector, including hospitals and regulatory bodies; and less than 2% are employed within pharmaceutical manufacturing [7]. This situation highlights the need for policies that will promote equitable distribution of pharmacists among different career domains to meet community needs. Choosing a career domain within the pharmacy profession is not a straightforward process. It is influenced by internal faculty-related factors, personal/family-related factors, and career domain-related factors [8]. Although the role of the pharmacy professional has expanded, it is often observed that pharmacy students do not select preferences until they have graduated. Increasing students’ awareness about future career planning could help to achieve goals in a successful manner.

Career planning and understanding the factors influencing career decisions are crucial to facilitate students’ improvement in the area they are interested in, and will be used in their future professional life. Globally, in the United States, the Accreditation Council for Pharmacy Education (ACPE) mentions the need for recruitment policies, as part of their document on accreditation standards [9]. In Sudan, Standards for Accreditation of Medical Schools (SAMS), which are based on World Federation for Medical Education (WFME) standards, include career guidance and planning as a quality development standard for accreditation of pharmacy schools [10]. Moreover, according to World Bank statistics, Sudan spends about 2.2% of its limited gross domestic product (GDP) on education [11].

There should be a well-thought-out link between education and career progression, particularly in pharmacy colleges, due to the high diversity of pharmacy career domains and high cost of pharmacy schools. The current situation in Sudan indicates that undergraduate students who do not determine their future career orientations before graduation are beginning to work in an unplanned way after graduation, and consequently, this results in a loss of interest, low productivity at work, or even failure if they choose a profession that is incompatible with their abilities. Thus, understanding the factors that influence undergraduate pharmacy students’ choice of a particular professional domain will help undergraduate pharmacy students develop an accurate perception of pharmacy profession domains and make information-based decisions about their career choice. This could enhance recruitment strategies, job satisfaction, and retention as well as productivity. Thus, the current study was carried out to identify job preferences and the decision-influencing factors among undergraduate pharmacy students at the University of Khartoum.

## 2. Materials and Methods

### 2.1. Study Design and Setting

A descriptive cross-sectional study was conducted at the Faculty of Pharmacy, University of Khartoum, Sudan. The Faculty of Pharmacy was established in 1964 and remained the only one in Sudan for about three decades. The study was conducted from March to May 2021.

### 2.2. Study Population

The study population was undergraduate pharmacy students. Only the third-, fourth-, and fifth-year students who were registered and undertook courses in the Bachelor of Pharmacy program during the study period were included in the study. First- and second-year students are not included in the study as in the first two years of the syllabus focus on basic medical and pharmaceutical science; thus, they have yet to establish enough awareness about pharmacy profession domains.

### 2.3. Sample Size and Sampling

The sample size was calculated using “Raosoft”, a sample size calculation software product, with 95% confidence intervals and a 5% margin of error with an expected response distribution of 50% [12]. Based on the data obtained from the Faculty of Pharmacy, the accessible study population was 454 students (third year: 180 students, fourth year: 95 students, and fifth year: 179 students). The minimum sample size required was 209 students (third year: 83 students, fourth year: 44 students, fifth year: 82 students). Two probability sampling methods were used to select the participants: stratified and systematic sampling. The study population was divided into three strata according to the academic year of study, and then a sample size appropriate to stratum size was obtained separately from each stratum using systematic sampling. The first unit of each stratum was randomly selected.

### 2.4. Data Collection

A self-administered structured questionnaire was used to collect data. The questionnaire was adapted from the previous studies undertaken using a questionnaire with confirmed reliability (pilot study) and internal consistency (Cronbach’s α > 0.7) [8], and it covered pharmacy students’ career preferences and factors influencing career choice [8,13]. The questionnaire consisted of three parts: the first part explored the socio-demographic characteristics of the participants; the second part contained one question and 12 options investigating the career domain preferred by students; and the third part consisted of 16 questions/items designed to access factors influencing future career domain choices. These factors were arranged into three themes: faculty-related influences (curriculum course/subject content, faculty extracurricular activities, a faculty member’s advice, and visits to a workplace); personal-related influences (family members’/relatives’ advice, a family member’s career choice, a friend’s career choice, good social status, and interaction with a practicing pharmacist); and job-related influences (opportunity for self-employment, an opportunity for part-time work, an opportunity for promotion and advancement, opportunity for health insurance, job salary and incentives, job allowances, and training in a workplace). A five-point Likert scale ranging from strongly agree to strongly disagree was used to rate the participants’ responses to the third part of the questionnaire. Two senior experts revised the questionnaire to ensure its validity. The questionnaire was also pre-tested with selected students to check the validity of the questions. Suggestions obtained from these experts and students were considered as amendments in preparing the final draft. The data from the pretest were not included in the final study.

A web-based Google form was used to create the online questionnaires that were automatically hosted via a unique uniform resource locator (URL). The URL link ensured the confidentiality of data and gave participants access from anywhere via their personal smartphone, laptop, or desktop computer. Preselected study participants were invited individually to participate through their contact information. Responses were collected from 23 March 2021 to 17 April 2021 and automatically sorted in a “Google Drive” database.

### 2.5. Data Analysis

The Statistical Package for Social Sciences (SPSS) version 26 software (IBM Corporation, Armonk, NY, USA) was used to analyze the data. The chi-square test was used to examine significant difference or association between independent socio-demographic variables (gender, year of study) and dependent variables. Data with a *p*-value of 0.05 or less were considered statistically significant.

## 3. Results

### 3.1. Demographic Characteristics of the Respondents

Out of 220 pharmacy students enrolled in the study, 189 (85.9%) were female. The average age of respondents was 21.7 ± 1.5 years, with a range of 19 to 29 years. About 38.6% of respondents were in the fifth year, 23.2% were in the fourth year, and 38.2% were in the third year of study.

Studying pharmacy was the first preferred choice for 161 (73.2%) of respondents at the time of application to universities, with insignificant associations between pharmacy as a first-preferred program, gender, and study year (Table 1).

### 3.2. The First-Choice Career Domain of Participants

Clinical pharmacy was selected as the most-preferred career domain after graduation (n: 64; 29.9%), followed by academia and research (n: 26; 11.8%), the pharmaceutical industry (n: 24; 10.9%), community pharmacy (n: 23; 10.5%), and public health (n: 14: 6.4%). Drug quality control, medical representatives, and drug regulatory bodies were marked as the least-preferred career domains by 10 (4.5%), 7 (3.2), and 1 (0.5%) of the respondents, respectively. Importantly, about 20% of participants preferred to move abroad for work. Moreover, data analysis revealed a significant association between gender and preferred career domain (*p*-value: 0.015) (Table 2).

### 3.3. Factors Influencing Future Career Choice

Factors influencing future career domains were broadly arranged into three categories: faculty-related factors, personal/family-related factors, and job-related factors. Out of five faculty-related factors, 178 (80.9%) of respondents strongly agreed or agreed that training in a workplace (pharmacy, industry, etc.) influenced career domain choice. Regarding personal/family-related factors, 158 (71.8%) and 140 (63.6%) of respondents strongly agreed or agreed that interaction with practicing pharmacists and good social status influenced career domain choice, respectively. On the other hand, 171 (77.7%) and 162 (63.6%) of the respondents either strongly agreed or agreed that job salary and job allowances, respectively, influenced career domain choice. Furthermore, chi-square analysis revealed that gender was insignificantly associated with influencing future career domain choice decisions (Table 3). Significant associations, with *p*-values of 0.024 and 0.017, were found between the influence of a family member’s career choice or interaction with practicing pharmacists, respectively, and the year of study (Table 4).

## 4. Discussion

Exploring students’ preferences toward different future career domains and their motivational variables is essential to designing and implementing future career orientation programs. To our knowledge, this is the first study that attempted to assess preferred career domains and factors influencing career domain choice decisions in Sudan. Results of this study highlighted the high female-to-male ratio (86%:14%). This finding is similar to those in many studies conducted among pharmacy students in Jordan [8,14]. Saudi Arabia [15], and Malaysia [16,17]. Since admission to the faculty is based on students’ academic achievement via the Sudanese secondary school certificate, and over 50% of admitted students are female, the high female-to-male ratio could be attributed to the fact that top-ranked female students prefer health sciences. Moreover, female students demonstrate higher academic achievement on Sudanese secondary school examinations than male students [18].

Nearly three-quarters of respondents indicated that studying pharmacy was their first-preferred choice. This result is similar to those concluded in studies conducted in South Africa [13] and the United Kingdom [19]. However, the current study finding is much higher than reported in studies conducted in Sierra Leone, where one-quarter of the respondents chose pharmacy as the first study field [20]. Furthermore, it is higher than the findings of a survey in Saudi Arabia, where about 40% of respondents selected the study of pharmacy as the first choice [21]. It is expected that students who attain high academic achievement in secondary school studies largely choose to study medicine as their first choice, with pharmacy or another health-related program as a second choice [13,22].

Regarding students’ future career domain of practice preference, our study showed that working as a clinical pharmacist was the most desired career domain (29.9%). The strong desire of respondents for practicing clinical pharmacy could be attributed to the ambition of students to keep pace with recent advancements in the pharmacy profession. In addition, the availability of work opportunities with good salaries, particularly abroad, may positively influence participants to prefer this field. In 2008, the American College of Clinical Pharmacy (ACCP) developed the core competencies of the clinical pharmacist. The proposed core competencies were, in brief: optimization of medication therapy, promotion of health, wellness, and disease prevention [23]. Fortunately, the B. Pharm curriculum was changed in 2016 from a traditional focus on pharmaceutical science courses to a modern curriculum that integrates more pharmacy practice and clinical pharmacy courses. Moreover, a clinical pharmacy training unit was established at Soba University Hospital [24]. Reform of the curriculum will help to produce future pharmacists with competency in providing patient care in collaboration with physicians and other health care providers. Currently, the level of clinical pharmacy services provided is low, and collaborative measures and support from health care professionals are needed to overcome the challenges and improve clinical pharmacy practice in Sudan. 

The second most preferred career domain was academia and research (11.8%), which differs from the results of Ethiopian [25] and South African [13] studies, where academia and research attracted 16% and 9.2% of respondents, respectively. Our findings, on the other hand, partially agree with those of studies conducted in Jordan [14], Saudi Arabia [15], and Australia [26], where academia and research are the most popular career paths. The main motivators toward academia and research include favorable opportunities for professional development, the chance to shape the future of pharmacy, the autonomy of the positions, and the flexible working environment. In addition, student participation in teaching and research via student-centered active learning may further attract students to this field [27].

The third-preference future career options for respondents were the pharmaceutical industry (10.9%) and community pharmacy (10.5%). This study’s findings are in parallel with the findings of a study conducted among Malaysian pharmacy students [16], but not in line with the findings of two studies conducted in Saudi Arabia, where they reported the pharmaceutical industry and community pharmacy as the least preferred career domains [15,28], or the findings of a study among Iraqi pharmacy students, where community pharmacy was ranked as the first-choice future career option [29]. This finding is significant because it contradicts the current distribution of the pharmacist workforce in these domains; according to literature, approximately 67% of pharmacists work in private community pharmacies, while less than 2% are employed in the pharmaceutical industry sector [30,31]. Relatively low preferences for community pharmacies could indirectly impact the reported low levels of job satisfaction among community pharmacists [7,32]. Since private pharmacies are the most available workplace for pharmacy graduates, efforts should be directed to design and apply policies to improve community pharmacy job satisfaction and performance. 

The study revealed that medical sales representative was an undesirable career domain, as it was chosen by only 3.2% of respondents. This finding contradicts the studies conducted in Jordan and Iraq [14,29], in which participants ranked medical representatives among the top-three preferred future career domains. Globally, most pharmaceutical companies allocate a relatively high budget for employing and training medical representatives; pharmaceutical product promotion and marketing expenditure is higher than research and development expenditure [33]. The negative attitude toward medical sales representatives could be an indirect consequence of the prolonged drug shortage in Sudan since the COVID-19 pandemic lockdown measures, further aggravated by local currency inflation [34], with the lockdown and economic instability making it challenging for many pharmaceutical companies to thrive.

The study also indicates that drug regulatory bodies were undesirable career domains, as they were chosen by only 0.5% of respondents. This finding was consistent with studies conducted in Malaysia [18], and Jordan [8,22], all of which found the drug regulatory affairs domain to be one of the least-preferred options. A drug regulatory body is a relatively new profession that governments have established to control the safety and efficacy of pharmaceutical products and medical devices [35]. The low preference of participants for some future career domains, such as drug regulatory bodies, may be due to a lack of sufficient knowledge and awareness about these career domains. The regular revision of the curriculum, providing career ordination, and workplace training programs are crucial to make students aware of various pharmacy profession opportunities, and the importance of their role in different career domains. 

As reported in other medical professions in Sudan, approximately 22% of participants wish to migrate, and these findings are not surprising, as reported in other medical professions in Sudan [35,36]. A global report pointed to a shortage of pharmacy professionals in Africa, particularly in low-income developing countries [37]. The massive brain-drain of health professions has a negative impact on services in the country. Therefore, efforts should be focused on managing migration [36]. 

Gender may affect the selection of the response to the most preferred career options. Sudan, as with other Arab communities, is a conservative society in which females prefer to work in a place with flexibility and fewer working hours. In the current study, a relatively large percentage of females preferred to work in academia and research (22.7%) and community pharmacy (11.6%), while the males’ preference for these domains was 6.5% and 3.2%, respectively. On the other hand, a high proportion of males showed a preference to working as medical sales representatives (13%), compared to females (1.6%). This finding agrees with reports from Jordan [8], and Saudi Arabia [38], where the influence of gender on future career choice was observed and attributed to cultural and social reasons [38]. Fieldwork, outstation trips, night shifts, and weekend hours that may be required to complete tasks, make the medical representative position more appealing to men [8].

When investigating the motivational factors behind the students’ choice of a particular pharmacy career domain, data analysis revealed that the key faculty-related factor was training in a workplace (around 51% strongly agree, 30% agree), followed by a curriculum/course at college (around 70% agree), which is consistent with a study conducted in Saudi Arabia reporting that previous training in a hospital and in community pharmacy had a significant impact on student future career choice [28]. This finding draws attention to the importance of workplace-based learning/training for students. Workplace training allows the student to apply their knowledge and gain social, cultural, and professional values; this implicit sort of workplace-based learning is known as the hidden curriculum, and has been identified as a significant issue in health professional education [39].

Personal/family-related influences and interaction with practicing pharmacists were ranked as the top factors by 72% (30% strongly agree, 42% agree), while a family member’s career choice and a friend’s career choice were ranked as the minor motivational factors (only 7.5% and 26% of participants respectively either agree or strongly agree). This finding contrasts with a study conducted in the United Arab Emirates, which reported the minimal influence of pharmacists as role models on students’ career selection [40]. In agreement with our findings, a study conducted in Saudi Arabia reported the influence of friends and family as minor motivational factors, at 16.5% and 18.5%, respectively [15]. Importantly, the association between the influence of training in a workplace and the year of study was statistically significant (*p*-value 0.01).

The job salary and incentives (78%), followed by the opportunity for promotion and advancement (75%), were the most important job-related factors influencing future career domain choice. This finding is consistent with studies from Saudi Arabia [15,28,41]. In contrast, a similar study in the United States concluded that the job environment was the most important factor influencing career decisions [42]. There was no significant association between socio-demographic characteristics and job-related factors (*p*-value > 0.005)

## 5. Limitations

There were some limitations to this study. It was conducted in one university; thus, it cannot be generalized to pharmacy students in other universities. It was also a cross-sectional study and administered to the students at one point in time. However, students’ choices may change with exposure to experiences; repeating the survey as students progress may enable evaluation of the consistency of students’ career choices and motivational factors. Additionally, the option ‘working outside Sudan’ was written under pharmacy career domains, not in a separate section. 

Despite these limitations, our study is novel as it is the first report that assessed the views of pharmacy students towards preferred career domains in Sudan. The findings of the study will inform and guide university authorities, Sudanese pharmaceutical societies, and other stakeholders about the factors that affect students in choosing a pharmacy career domain. It is recommended that the gap between the implemented curriculum and employment skills should be narrowed through auditing and regular updates of the curriculum. In addition, establishing training programs in collaboration with governmental and private bodies can further increase students’ awareness about career domains. Moreover, organizing activities, such as career days, symposiums, and workshops, for various areas of pharmacy professions, particularly for arising career domains, will enable students to identify and achieve their future career goals.

## 6. Conclusions

The present study highlighted a baseline understanding of the career preference and main factors influencing future career domain choice among undergraduate pharmacy students at the University of Khartoum. The study showed a positive attitude in most students towards pharmacy when applying to the program. Clinical pharmacy, academia and research, the pharmaceutical industry, and community pharmacy were the most preferred choices of students. The main factors that influenced career preference were training in a workplace, curriculum content, interaction with practicing pharmacists, job salary and incentives, and the opportunity for promotion and advancement.

## Figures and Tables

**Table 1:** Participants’ choice of pharmacy as the first-preferred program in association with their demographic characteristics.

Characteristic		Yes		No		*p*-Value
		Frequency	Percentage	Frequency	Percentage	
Gender	Female (n: 189)	141	74.6	48	25.4	0.169
	Male (n: 31)	20	64.5	11	35.5	
	Total (n: 220)	161	73.2	59	26.8	
Study year	Third-year (n: 85)	66	77.6	19	22.4	0.664
	Fourth-year (n: 51)	35	68.6	16	31.4	
	Fifth-year (n: 84)	60	71.4	24	28.6	
	Total (220)	161	73.2	59	26.8	

**Table 2:** Association between preferred career domain and demographic characteristics.

Variable	Career Domain/Response											*p*-Value
	Aca * & Res.	Clin Ph.	Com. Ph.	Drug Q.C	Drug Reg.	Med. Rep.	Pha. Ind.	Pub. Hea.	W. Out	No. W.	Other	
**Gender:**												
Female(N)	24	41	22	8	0	3	21	13	40	2	15	0.015 **
Female (%)	12.7	21.7	11.6	4.2	0.0	1.6	11.1	6.9	21.2	1.1	7.9	
Male (N)	2	5	1	2	1	4	3	1	9	0	3	
Male (%)	6.5	16.1	3.2	6.5	3.2	12.9	9.7	3.2	29.0	0.0	9.7	
**Year of study:**												
3th (N)	13	14	10	6	1	4	10	5	15	0	7	0.724
3th (%)	15.3	16.5	11.8	7.1	1.2	4.7	11.8	5.9	17.6	0.0	8.2	
4th (N)	5	14	4	2	0	1	7	3	13	0	2	
4th (%)	9.8	27.5	7.8	3.9	0.0	2.0	13.7	5.9	25.5	0.0	3.9	
5 th (N)	8	18	9	2	0	2	7	6	21	2	9	
5 th (N)	9.5	21.4	10.7	2.4	0.0	2.4	8.3	7.1	25.0	2.4	10.7	

* Aca & Res.: Academia and research, Clin. ph.: Clinical pharmacy, Com. ph.: Community pharmacy, Drug Q.C., Drug quality control, Drug reg.: drug regulatory bodies, Med. rep.: Medical representatives, Pha. ind.: Pharmaceutical industry, Pub. Hea.: Public health, W. out.: Working outside Sudan, No. w.: Not working. ** Significant difference between the compared groups at *p*-value < 0.05.

**Table 3:** Association between gender and different factors influencing career choice.

Factor	Response/Gender										*p*-Value
	Strongly Agree (%)		Agree (%)		Neutral (%)		Disagree (%)		Strongly Disagree (%)		
	F	M	F	M	F	M	F	M	F	M	
**Faculty-related influences:**											
Curriculum course/subject content	28.0	29.0	42.9	48.4	23.8	19.4	4.8	3.2	0.5	0.0	0.413
Faculty extracurricular activities	23.8	32.3	41.8	38.7	26.5	29.0	4.2	0.0	3.7	0.0	0.775
Faculty member advice	18.5	12.9	47.1	67.7	25.4	6.5	7.9	0.0	1.1	12.9	0.216
Visits to a workplace	42.3	38.7	34.4	38.7	15.3	22.6	4.8	0.0	3.2	0.0	0.481
Training in a workplace	51.3	51.6	28.6	35.5	11.6	9.7	5.3	3.2	3.2	0.0	0.885
**Personal-related influences:**											
Family members’/relatives’ advice	14.3	16.1	28.0	54.8	38.6	22.6	11.1	0.0	7.9	6.5	0.885
A family member career choice	4.8	12.9	21.7	22.6	34.9	35.5	21.7	9.7	16.9	19.4	0.885
A friend’s career choice	5.3	6.5	18.5	38.7	32.8	25.8	28.6	12.9	14.8	16.1	0.116
Good social status	25.4	29.0	37.0	41.9	24.9	25.8	8.5	0.0	4.2	3.2	0.634
Interaction with practicing pharmacist	31.7	16.1	42.9	61.3	18.0	16.1	5.8	6.5	1.6	0.0	0.501
**Job-related influences:**											
Opportunity for self-employment	27.0	22.6	46.0	54.8	16.9	16.1	7.9	3.2	2.1	3.2	0.763
Opportunity for part-time work	22.2	16.1	39.7	48.4	27.5	29.0	9.0	0.0	1.6	6.5	0.199
Opportunity for promotion and advancement	28.6	22.6	45.5	64.5	20.6	12.9	3.7	0.0	1.6	0.0	0.409
Opportunity for health insurance	26.5	16.1	42.3	58.1	24.3	22.6	4.8	0.0	2.1	3.2	0.404
Job salary and incentives	37.0	38.7	38.6	54.8	16.4	0.0	5.8	0.0	2.1	6.5	0.163
Job allowances (car, house)	36.5	41.9	33.9	54.8	22.8	3.2	4.8	0.0	2.1	0.0	0.201

**Table 4:** Association between the year of study and different factors influencing career choice.

Factors	Response/Year of Study															*p*-Value
	Strongly Agree (%)			Agree (%)			Neutral (%)			Disagree (%)			Strongly Disagree (%)			
	3rd	4th	5th	3rd	4th	5th	3rd	4th	5th	3rd	4th	5th	3rd	4th	5th	
**Faculty-related influences:**																
Curriculum course/subject content	27.1	29.4	28.6	41.2	49.0	42.9	28.2	19.6	20.2	3.5	2.0	7.1	0.0	0.0	1.2	0.819
Faculty extracurricular activities	23.5	29.4	23.8	44.7	35.3	41.7	27.1	31.4	23.8	2.4	3.9	4.8	2.4	0.0	6.0	0.71
Faculty member advice	17.6	25.5	13.1	48.2	43.1	56.0	23.5	23.5	21.4	7.1	7.8	6.0	3.5	0.0	3.6	0.622
Visits to a workplace	55.3	39.2	29.8	28.2	31.4	44.0	12.9	21.6	16.7	2.4	7.8	3.6	1.2	0.0	6.0	0.014
Training in a workplace	60.0	52.9	41.7	24.7	23.5	38.1	8.2	11.8	15.5	5.9	9.8	0.0	1.2	2.0	4.8	0.01 *
**Personal-related influences:**																
Family members’/relatives’ advice	14.1	13.7	15.5	38.8	33.3	26.2	35.3	35.3	35.7	9.4	7.8	13.1	2.4	9.8	9.5	0.693
A family member’s career choice	8.2	9.8	2.4	25.9	11.8	23.8	43.5	25.5	32.1	12.9	27.5	22.6	9.4	25.5	19.0	0.024 *
A friend’s career choice	4.7	11.8	2.4	20.0	23.5	21.4	42.4	21.6	27.4	24.7	29.4	26.2	8.2	13.7	22.6	0.074
Good social status	24.7	33.3	22.6	44.7	39.2	29.8	25.9	13.7	31.0	3.5	9.8	9.5	1.2	3.9	7.1	0.13
Interaction with practicing pharmacist	37.6	27.5	22.6	41.2	49.0	42.9	18.8	23.5	11.9	2.4	7.8	8.3	0.0	0.0	4.8	0.017 *
**Job-related influences:**																
Opportunity for self-employment	27.1	29.4	23.8	49.4	43.1	47.6	17.6	15.7	16.7	4.7	11.8	7.1	1.2	2.0	4.8	0.67
Opportunity for part-time work	23.5	27.5	15.5	44.7	31.4	42.9	23.5	29.4	31.0	7.1	9.8	7.1	1.2	2.0	3.6	0.555
Opportunity for promotion and advancement	30.6	31.4	22.6	44.7	41.2	56.0	21.2	23.5	15.5	3.5	3.9	2.4	0.0	0.0	3.6	0.222
Opportunity for health insurance	34.1	25.0	16.7	43.5	36.5	50.0	17.6	32.7	25.0	3.5	3.8	4.8	1.2	1.9	3.6	0.151
Job salary and incentives	40.0	39.2	33.3	36.5	35.3	48.8	10.6	21.6	13.1	8.2	3.9	2.4	4.7	0.0	2.4	0.134
Job allowances (car, house)	40.0	35.3	35.7	40.0	27.5	39.3	10.6	33.3	21.4	7.1	3.9	1.2	2.4	0.0	2.4	0.074

* Significant difference between the compared groups at *p*-value < 0.05.

## Data Availability

All data used and analyzed during the current study are available from the corresponding author on reasonable request.
